# Inflammatory risk contributes to post-COVID endothelial dysfunction through anti-ACKR1 autoantibody

**DOI:** 10.26508/lsa.202402598

**Published:** 2024-05-13

**Authors:** Ee-Soo Lee, Nhi Nguyen, Barnaby E Young, Hannah Wee, Vanessa Wazny, Khang Leng Lee, Kai Yi Tay, Liuh Ling Goh, Florence WJ Chioh, Michelle CY Law, I. Russel Lee, Lay Teng Ang, Kyle M Loh, Mark Y Chan, Bingwen E Fan, Rinkoo Dalan, David C Lye, Laurent Renia, Christine Cheung

**Affiliations:** 1 Lee Kong Chian School of Medicine, Nanyang Technological University , Singapore, Singapore; 2 Yong Loo Lin School of Medicine, National University of Singapore, Singapore, Singapore; 3 Department of Infectious Diseases, Tan Tock Seng Hospital, Singapore, Singapore; 4 National Centre for Infectious Diseases, Singapore, Singapore; 5 Department of Endocrinology, Tan Tock Seng Hospital, Singapore, Singapore; 6 Stanford Institute for Stem Cell Biology and Regenerative Medicine, Stanford University, Stanford, CA, USA; 7 Department of Developmental Biology, Stanford Cardiovascular Institute, Stanford University, Stanford, CA, USA; 8 National University Heart Centre, National University Health System, Singapore, Singapore; 9 Department of Haematology, Tan Tock Seng Hospital, Singapore, Singapore; 10 Department of Laboratory Medicine, Khoo Teck Puat Hospital, Singapore, Singapore; 11 A*STAR Infectious Diseases Labs, Agency for Science, Technology and Research, Singapore, Singapore; 12 Institute of Molecular and Cell Biology, Agency for Science, Technology and Research, Singapore, Singapore

## Abstract

Subclinical vascular impairment can be exacerbated in individuals who experience sustained inflammation after COVID-19 infection. Our study explores the prevalence and impact of autoantibodies on vascular dysfunction in healthy COVID-19 survivors, an area that remains inadequately investigated. Focusing on autoantibodies against the atypical chemokine receptor 1 (ACKR1), COVID-19 survivors demonstrated significantly elevated anti-ACKR1 autoantibodies, correlating with systemic cytokines, circulating damaged endothelial cells, and endothelial dysfunction. An independent cohort linked these autoantibodies to increased vascular disease outcomes during a median 6.7-yr follow-up. We analyzed a single-cell transcriptome atlas of endothelial cells from diverse mouse tissues, identifying enriched *Ackr1* expressions in venous regions of the brain and soleus muscle vasculatures, which holds intriguing implications for tissue-specific venous thromboembolism manifestations reported in COVID-19. Functionally, purified immunoglobulin G (IgG) extracted from patient plasma did not trigger cell apoptosis or increase barrier permeability in human vein endothelial cells. Instead, plasma IgG enhanced antibody-dependent cellular cytotoxicity mediated by patient PBMCs, a phenomenon alleviated by blocking peptide or liposome ACKR1 recombinant protein. The blocking peptide uncovered that purified IgG from COVID-19 survivors possessed potential epitopes in the N-terminal extracellular domain of ACKR1, which effectively averted antibody-dependent cellular cytotoxicity. Our findings offer insights into therapeutic development to mitigate autoantibody reactivity in blood vessels in chronic inflammation.

## Abstract (precis)

We study the prevalence and impact of autoantibodies on vascular dysfunction in healthy COVID-19 survivors, revealing elevated anti-ACKR1 autoantibodies associating with systemic cytokines and endothelial dysfunction. Plasma IgG enhances cellular cytotoxicity, suggesting therapeutic avenues to mitigate vascular autoantibody reactivity post-infection.

## Graphical Abstract

**Figure:**
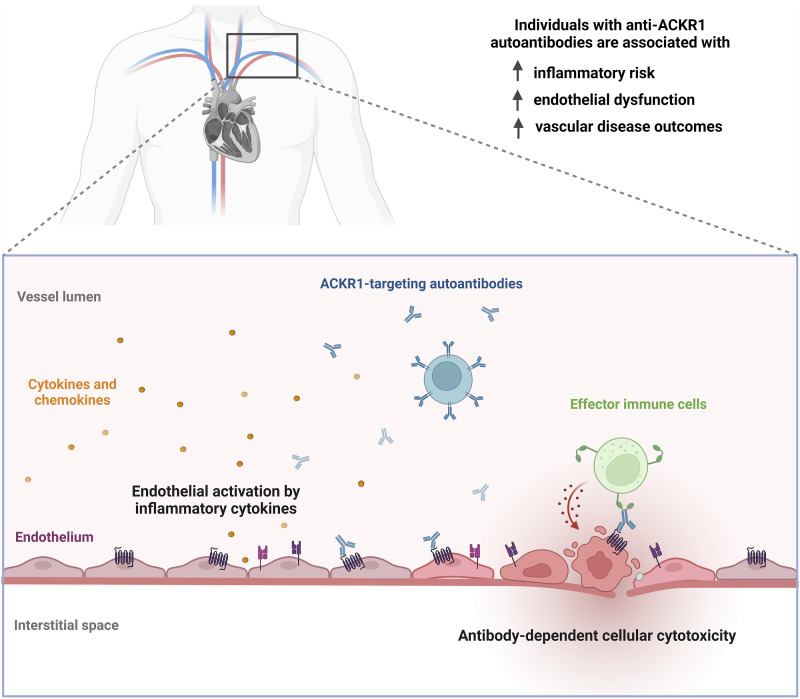

## Introduction

The incidence of adverse vascular events in COVID-19 survivors declines with time after the acute infection phase. However, the relative risk within the whole population of thrombotic complications remains elevated for up to 49 wk post-COVID-19 diagnosis (1). An emerging consensus highlights chronic low-grade inflammation as a pathological driver of vascular dysfunctions in commonly co-occurring cardiometabolic conditions. These can be aggravated by the persistent elevation of proinflammatory cytokines and immunological anomalies observed in some post-COVID-19 individuals (2). Hence, a single therapeutic approach may not be sufficient for optimal management of inflammatory and atherothrombotic disease states.

In our pursuit for alternative targets, we directed our attention to the immunological drivers of vascular dysfunctions. Our prior studies on COVID-19 survivors revealed detectable endotheliopathy in convalescent patients (3), persisting even ∼1 yr after recovery in individuals (4). Proinflammatory cytokines and activated T-lymphocyte-associated factors stemming from SARS-CoV-2 infection were found to be associated with inflammatory activation markers on damaged endothelial cells in these patients (3). It is noteworthy that endothelial cell activation and antigen presentation can occur in various diseases, including autoimmune disorders, chronic inflammation, and infections. In such cases, endothelial cells play a role in driving immune responses, leading to the production of T helper type-1 cytokines and T-cell-mediated cytotoxicity (5, 6, 7). Furthermore, autoantibodies have been identified in a diverse range of conditions associated with cardiovascular pathologies, including systemic vasculitis (8, 9), atherosclerosis (10), dilated cardiomyopathy, and valvular heart disease (11). It has been proposed that autoantibodies targeting specific endothelial cell antigens may contribute to blood vessel injuries (9, 12). These observations underscore the importance of considering autoimmune mechanisms in the currently underdiagnosed vasculopathy when managing patients with cardiovascular risk factors.

Relevantly, COVID-19 has been shown to stimulate autoantibody reactivities against immunomodulatory proteins and vascular tissues, concurrently impairing immunoreceptor signaling and exacerbating viral pathogenesis (13, 14). Whereas autoantibodies have been identified in infected patients (2), their prevalence post-recovery and their impact on subclinical vascular impairment in overall healthy populations remain inadequately investigated. Our hypothesis speculates that chronic inflammation induces vascular endothelial cells to acquire antigen-presenting characteristics, thereby leading to the production of autoantibodies targeting endothelial components. Putative mechanisms involved in autoantibody-mediated vascular damage encompass endothelial inflammatory activation, induction of apoptosis, as well as antibody-dependent cell cytotoxicity and complement-dependent cytotoxicity (8, 9). Autoantibodies reacting with tissue-associated antigens showed connections with distinct clinical features. Here, our study aimed to identify specific autoantibodies targeting endothelial cell antigens, elucidate the mechanisms underpinning autoantibody-mediated vascular damage, and explore how these targets contribute to individuals’ risks of vascular dysfunction. The modulation of interactions between endothelial cells and immunological drivers may offer a potential avenue for restoring vascular health.

## Results and Discussion

### Tissue and cell type expressions of endothelial antigens

**Figure S1.**
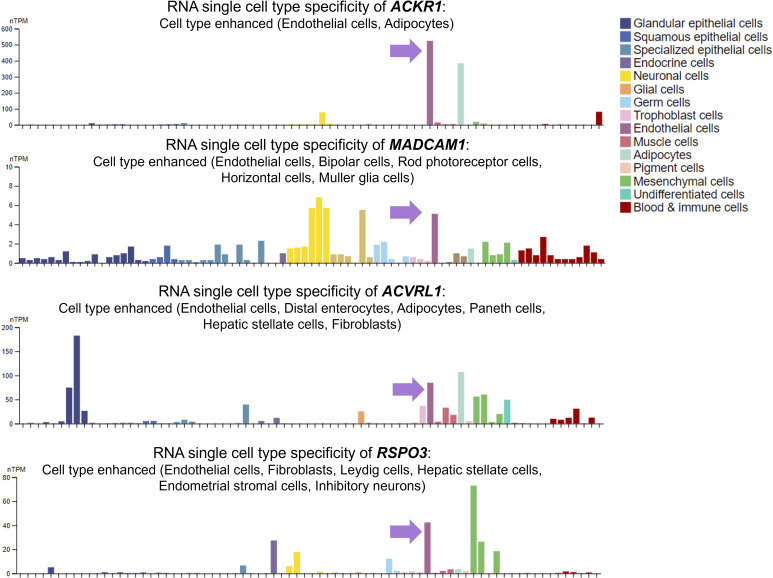
Cell type specificity of endothelial antigens. Normalized single-cell RNA (nTPM) from all single-cell types extracted from the Human Protein Atlas. Color-coding is based on cell type groups.

**Figure 1.**
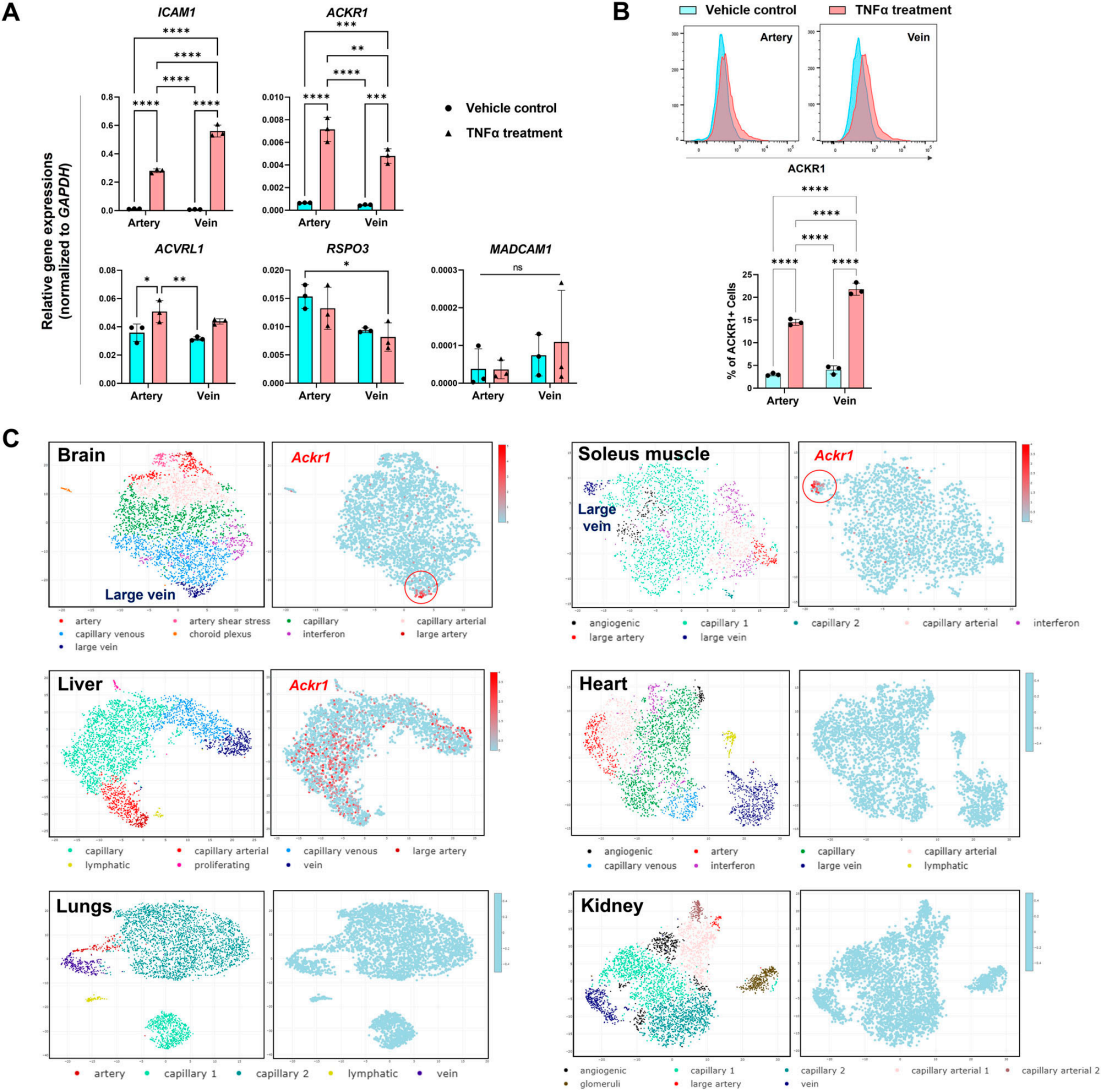
Tissue and cell type expressions of endothelial antigens. **(A)** Gene expressions of endothelial antigens upon TNFα (10 ng/ml) treatment on human umbilical artery and vein cells for 24 h. Data represent mean ± s.d.; n = 3 biological replicates; two-way ANOVA; *P < 0.05, **P < 0.01, ***P < 0.001, and ****P < 0.0001. **(B)** Flow cytometry analysis of ACKR1 protein expression on artery and vein cells after 48 h of stimulation with TNFα (10 ng/ml). Data represent mean ± s.d.; n = 3 biological replicates; two-way ANOVA; ****P < 0.0001. **(C)** Expression profile of *Ackr1* in vascular endothelial subtypes of various organs from a mouse single-cell transcriptome atlas (accession code: E-MTAB-8077). Data were generated with EC Atlas web-based visualization.

Infection with SARS-CoV-2 elicits the production of autoantibodies targeting immunomodulatory proteins and various tissues (13). Our analysis into the tissue-specific targets revealed several vascular antigens, including atypical chemokine receptor 1 (*ACKR1*), activin A receptor-like type 1 (*ACVRL1*), R-spondin 3 (*RSPO3*) and mucosal vascular addressin cell adhesion molecule 1 (*MADCAM1*). The cell type specificity of these antigens suggested enriched gene expressions in endothelial cells (Fig S1). Then, we leveraged human umbilical artery and vein cells for further gene expression profiling. To investigate the inducibility of these endothelial antigens under inflammatory conditions, we treated the artery and vein cells with recombinant human TNFα protein for 24 h. TNFα treatment led to endothelial activation, as evidenced by a significant up-regulation of *ICAM1* expression (Fig 1A). Interestingly, both artery and vein cells demonstrated substantial increase in *ACKR1* expressions, unlike the other endothelial antigens. ACKR1 is a member of the ACKR family, characterized by its lack of intracellular signaling transmission. In contrast to the conventional role of scavenging chemokines, ACKR1 exclusively facilitates chemokine transcytosis across endothelial layers, resulting in the apical presentation of chemokines to mediate leukocyte transmigration (15, 16). The expression of ACKR1 can be regulated by NF-κB and has been shown to be induced upon exposure to whole human blood, primarily attributed to interactions with neutrophils (17). Given that ACKR1 plays a pivotal role in inflammatory responses (18), we prioritized ACKR1 as the antigen of interest for further interrogation.

As TNFα is known to influence NF-κB activity, we studied the protein expression of ACKR1 in artery and vein cells exposed to recombinant human TNFα. After a 48-h treatment, we observed significant up-regulation of ACKR1 expressions in both vein and artery cells (Fig 1B). In the arteriovenous axis, the expression of ACKR1 is specifically confined to venular endothelial cells, excluding arterioles and capillaries (15, 19, 20, 21, 22). Here, even though there was a notable increase in the proportion of ACKR1-expressing vein cells compared with artery cells upon TNFα treatment, both vein and artery cells exhibited comparable baseline levels of ACKR1 expressions (Fig 1B), contradicting the expected venous specificity associated with ACKR1. This observation may be linked to the phenomenon where most primary endothelial cells tend to lose their subtype specificity following sub-culturing in vitro (23).

To better understand in vivo tissue-specific expressions of *Ackr1*, we conducted a computational analysis using a published single-cell transcriptome atlas of organotypic endothelial cells isolated from various mouse tissues (24). The data revealed enriched venous expressions of *Ackr1* in the brain and soleus muscle, as well as diffuse expressions of *Ackr1* across all endothelial subtypes in the liver (Fig 1C). However, negligible *Ackr1* expression was found in the heart, lungs, and kidney, etc. This distinctive tissue-specific expression pattern of *Ackr1* raised intriguing connections with venous thromboembolism manifestations reported in COVID-19 survivors, such as intracranial venous thrombosis and lower limb deep venous thrombosis (1, 25, 26). The organ- and tissue-specific clinical outcomes may suggest immunological attacks on specific tissue-associated antigens.

### Association of anti-ACKR1 autoantibodies with inflammatory risks, endothelial dysfunction, and incidence of future vascular outcomes

Given the involvement of ACKR1 in chemokine-driven responses, particularly in blood vessels (16), we postulated the presence of autoantibodies targeting ACKR1-expressing endothelial cells in individuals at risk of chronic inflammation. Using COVID-19 survivors as a reference group (demographic details in Table S1), we enrolled 38 individuals with a median age of 43 yr (interquartile range: 37–55), of whom 71% were male. A majority was infected with pre-delta variants (73.7%). The time interval post-hospitalization averaged 332 ± 96 d. These subjects recovered from asymptomatic (28.9%), mild (47.4%) and severe (23.7%) conditions. Of these subjects, two had a history of acute myocardial infarction, and one exhibited chronic diabetic vasculopathy. In conjunction, 27 uninfected controls (median age 55 yr, interquartile range: 49.5–60.5) comprise 74.1% males. No participants had a prior medical history involving stroke, venous thromboembolism, autoimmune disorders, or immunodeficiency.

Table S1 Demographics of COVID-19 survivors and non-infected controls.

**Figure 2.**
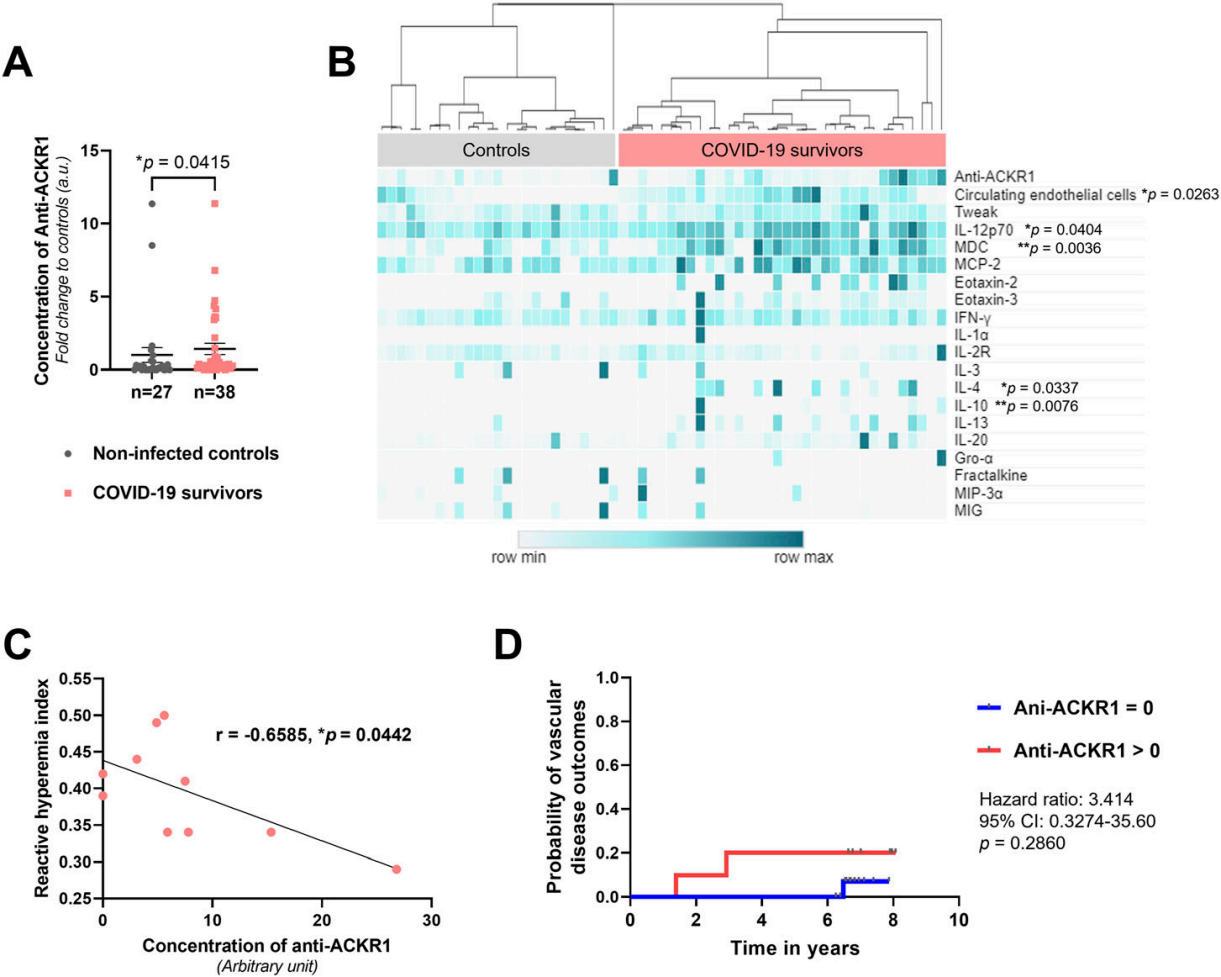
Association of anti-ACKR1 autoantibodies with inflammatory risks, endothelial dysfunction, and incidence of future vascular outcomes. **(A)** Scatterplot of the plasma levels of anti-ACKR1 autoantibodies in COVID-19 survivors and non-infected controls (mean ± s.e.m., Mann–Whitney test). Anti-ACKR1 concentrations were calculated based on normalized ratio over positive control of human immunoglobulin G (50 μg/ml) and presented as fold change to non-infected controls. **(B)** Heatmap depicting plasma concentrations of anti-ACKR1 autoantibodies, cytokines (quantified by Luminex multiplex assay), and enumeration of circulating endothelial cells in COVID-19 survivors and non-infected controls. Spearman’s correlation analysis revealed positive associations between anti-ACKR1 levels and the quantity of circulating endothelial cells, as well as each cytokine listed, with significant *P*-values indicated. Heatmap was generated using the MORPHEUS visualization software. **(C)** Spearman’s correlation analysis between anti-ACKR1 levels and reactive hyperemia index in individuals (n = 10) with endothelial dysfunctions (characterized by natural log-transformed reactive hyperemia index < 0.51). Spearman’s correlation coefficient r and *P*-values (two-tailed test) are indicated. **(D)** Kaplan-Meier plot shows the probability of primary vascular disease outcomes in patients without prior established cardiovascular diseases in a median follow-up period of 6.7 yr. Hazard ratio and 95% confidence intervals (CI) compare the time of blood collection to the first occurrence of vascular outcomes according to the presence of anti-ACKR1 autoantibodies (n = 16 undetectable anti-ACKR1, n = 10 anti-ACKR1 > 0 μg/ml).

**Figure S2.**
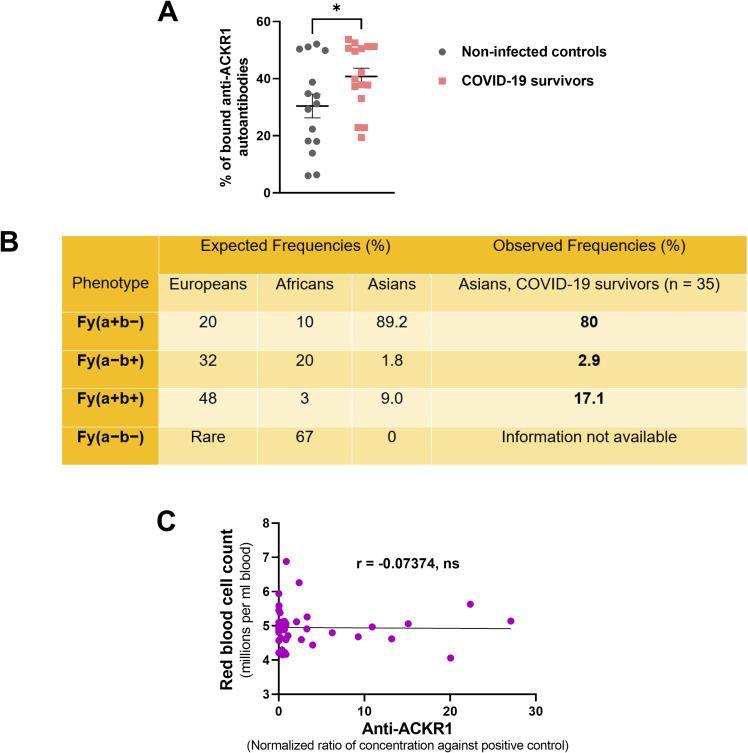
Functional impact of anti-ACKR1 autoantibodies on erythrocytes. **(A)** Scatterplot of the plasma levels of anti-ACKR1 autoantibodies in COVID-19 survivors and non-infected controls (mean ± s.e.m., Mann–Whitney test). Anti-ACKR1 levels were determined based on flow cytometry detection of bound anti-ACKR1 to K562 cells, a human erythroleukemic cell line ectopically overexpressing ACKR1. **(B)** ACKR1 (DARC) polymorphism, G125A (rs12075) was genotyped to identify individuals carrying FYA (G125) and FYB (125A) alleles, indicated under “Observed Frequencies.” “Expected frequencies” were extracted from literature (43) as a comparison. **(C)** Spearman’s correlation analysis to assess the associations between the circulating levels of anti-ACKR1 autoantibodies and red blood cell count (n = 46).

To measure anti-ACKR1 autoantibodies in human plasma, we designed a custom microarray-based kit to detect autoantibodies binding to immobilized recombinant ACKR1 protein on glass slides. Significantly elevated levels of anti-ACKR1 autoantibodies were observed in COVID-19 survivors compared with non-infected controls (Fig 2A). Because of potential limitations inherent in microarray-based detection, which used recombinant ACKR1 protein devoid of its native three-dimensional structure, we sought to validate our findings through an alternative approach. We conducted flow cytometry-based detection using K562 cells, a human erythroleukemic cell line ectopically overexpressing ACKR1. This would ensure the presence of posttranslational modifications crucial for maintaining protein conformation and proper cellular localization. We replicated the results demonstrating significantly higher levels of bound anti-ACKR1 autoantibodies in COVID-19 survivors than non-infected controls (Fig S2A).

Next, we assessed systemic inflammation using Luminex multiplex cytokine assays on plasma samples from the enrolled subjects. Positive correlations emerged between anti-ACKR1 and several cytokines, implicating a connection between proinflammatory factors and generation of anti-ACKR1 autoantibodies (Fig 2B). Certain COVID-19 survivors also exhibited heightened interleukin and chemokine levels. To explore the potential impact of anti-ACKR1 autoantibodies on endothelial dysfunction, we concurrently quantified circulating endothelial cells in their PBMC fractions. Circulating endothelial cells can be found in the bloodstream, representing a population of dysfunctional endothelial cells that are dislodged from damaged blood vessels (27, 28). Indeed, we observed a significant positive correlation between anti-ACKR1 autoantibody levels and circulating endothelial cell counts (Fig 2B), implicating vascular injury with the presence of anti-ACKR1 autoantibodies.

Quantitative evaluation of endothelial function using peripheral tonometry, specifically the reactive hyperemia index (RHI), serves as a robust surrogate marker for cardiovascular health. This method exhibits strong correlations with the Framingham Risk Score and is linked to delayed cardiovascular adverse events, including cardiovascular death or myocardial infarction (29). Upon analyzing individuals with abnormal RHI values (denoted as natural log–transformed RHI < 0.51), we observed a notable and statistically significant negative correlation with anti-ACKR1 levels (Fig 2C). These findings suggest important associations between anti-ACKR1 levels and endothelial dysfunction. Analyzes based on functional and humoral biomarkers, i.e., RHI and circulating damaged endothelial cells, respectively, showed that anti-ACKR1 could impact on early vascular alterations.

The use of pharmacological thromboprophylaxis as recommended for severe COVID-19 (30) may have limited our ability to establish the causal links between anti-ACKR1 autoantibodies and adverse vascular events. Therefore, we turned to an independent cohort characterized by prevalent cardiometabolic risks (demographic details in Table S2), where retrospectively biobanked plasma samples could be used for anti-ACKR1 measurement. After a median follow-up period of 6.7 yr, the incidence rate for primary composite vascular disease endpoint—encompassing nonfatal myocardial infarction, angina, heart failure, nonfatal stroke, transient ischemic attack, peripheral arterial disease, or vascular death, was generally elevated in the group possessing detectable anti-ACKR1 autoantibodies (Fig 2D). Although statistical significance was not reached because of the limited sample size, these findings suggest a greater likelihood of vascular disease outcomes among individuals with anti-ACKR1 autoantibodies compared with those without (hazard ratio 3.414; 95% confidence interval [0.3274–35.60]). We acknowledge that causal links between ACKR1 autoantibodies and adverse vascular events will require more extensive cohort studies.

Table S2 Demographics of independent cohort characterized by prevalent cardiometabolic risks without established cardiovascular diseases.

In addition, ACKR1 functions as an antigen in the Duffy blood group system, expressed not only in endothelial cells but also in erythrocytes. Among our COVID-19 survivors, 80% possessed the Fy(a+b−) phenotype (Fig S2B), in consensus with the high prevalence of FY*A/*A genotype in Asians. Despite the potential for autoantibodies targeting Duffy antigens to induce red blood cell destruction, anti-ACKR1 levels did not exhibit a significant correlation with red blood cell count (Fig S2C). Particularly, two participants with prior blood transfusions were negative for antibody screen, ruling out the development of red blood cell alloantibodies. Whereas antibodies against Duffy antigens can cause immune reactions, such as agglutination, they generally do not directly activate the complement cascade (31). The hemolytic reactions caused by Duffy antibodies are often mediated by other mechanisms, such as opsonization and phagocytosis by macrophages, or direct binding of antibodies to the red blood cells leading to their destruction. In our study, anti-ACKR1 autoantibodies were not found to be associated with hemolysis.

Endogenous expression of ACKR1 on endothelial cells seems to remain unaffected by genetic variations. Duffy-negative individuals of African descent often carry an *ACKR1* gene variant. Notably, the expression of ACKR1 antigens has been identified in the endothelial cells of Fy(a−b−) individuals who lack the receptor on their erythrocytes (21). Intriguingly, a single nucleotide polymorphism in the *ACKR1* gene, affecting the binding of the GATA1 transcription factor to the gene promoter, hinders the expression of the Duffy antigen on erythrocytes but does not impact its ACKR1 expression on endothelial cells (32). In this context, mechanistic studies to intervene the autoantigen-antigen interaction could be an effective strategy over gene silencing.

### Anti-ACKR1 autoantibodies induce endothelial dysfunctions

**Figure 3.**
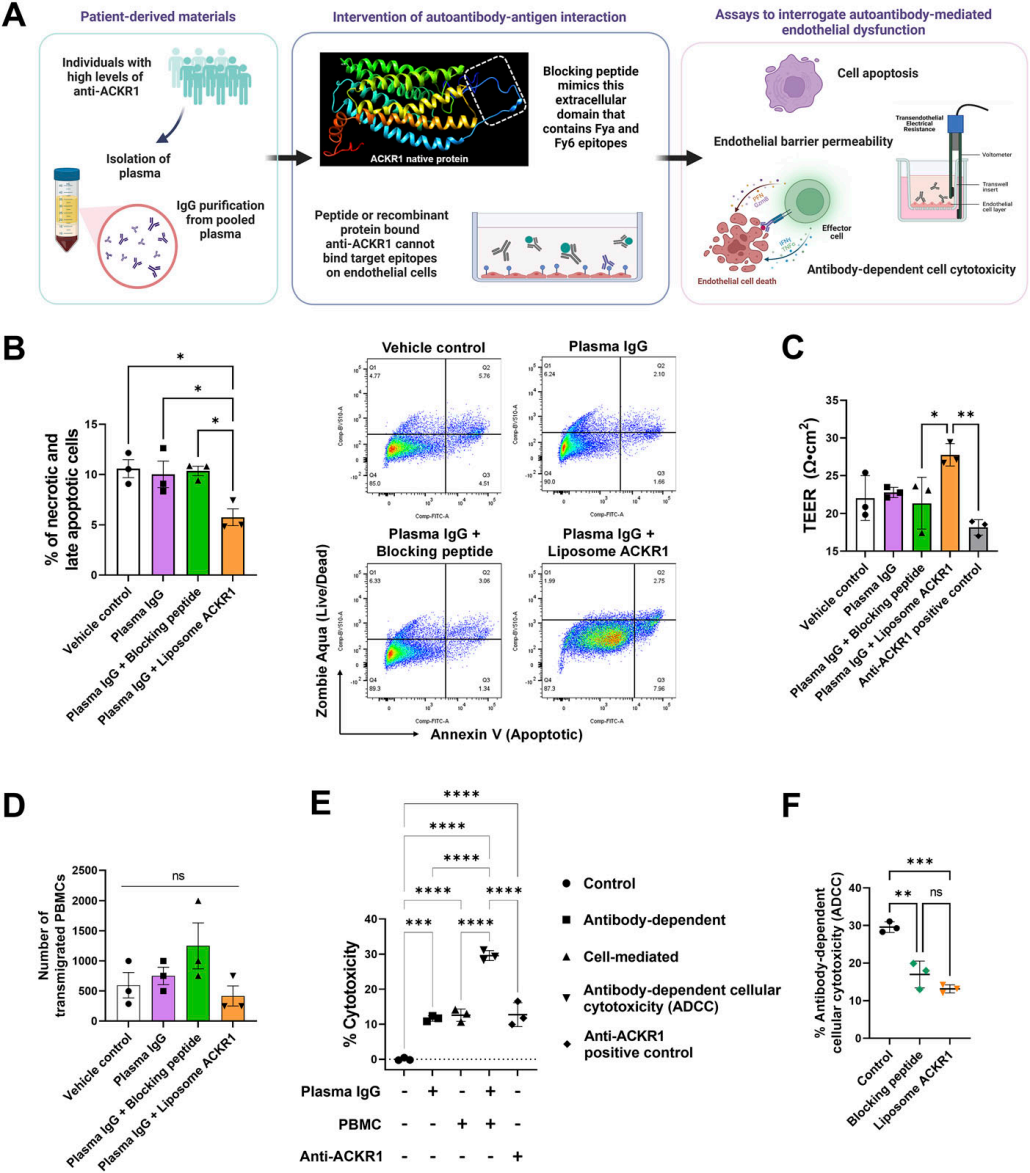
Anti-ACKR1 autoantibodies induce endothelial dysfunctions. **(A)** Experimental workflow of intervening autoantibody-antigen interactions to investigate the functional impact of anti-ACKR1 autoantibodies on human endothelial cells. The three-dimensional structure of ACKR1 was produced using I-TASSER server. **(B)** Flow cytometry analysis of the percentage of necrotic and late apoptotic endothelial cells after 24 h of experimental treatments. **(C)** Transendothelial electrical resistance assay to evaluate endothelial barrier permeability after experimental treatments. **(D)** Number of transmigrated PBMCs in an endothelial-immune cell co-culture transwell assay. **(E)** Degree of cytotoxicity was determined by quantifying lactate dehydrogenase activity in cell-free supernatants obtained from endothelial cells cultured with purified immunoglobulin G (IgG) and/or PBMCs. **(F)** Degree of antibody-dependent cellular cytotoxicity was determined from endothelial cells exposed to purified IgG and PBMCs. Purifie IgG was pre-incubated with and without blocking peptide or liposome ACKR1. For (B, C, D, E, F), data represent mean ± s.d.; one-way ANOVA; *P < 0.05, **P < 0.01; ***P < 0.001, ****P < 0.0001; ns, non-significant. Data points represent biological replicates.

We devised our experimental workflow to investigate the impact of anti-ACKR1 autoantibodies on human endothelial cells, examining how this effect could be mitigated by blocking peptides or recombinant proteins (Fig 3A). ACKR1, a seven-transmembrane glycoprotein, features multiple binding sites for chemokines and antibodies across its four extracellular domains (33). The blocking peptide, a synthetic 18 amino acid construct situated near the N-Terminus, located within the first 50 amino acids of human ACKR1, may target the common Fya and Fyb alleles. Another Fy6 epitope is also found within this region.

First, we pooled plasma samples (n = 5) with the highest levels of anti-ACKR1 among the COVID-19 survivors and isolated the immunoglobulin G (IgG) fraction. To attribute the endothelial dysfunctions to anti-ACKR1 autoantibodies, we pre-incubated purified IgG with a blocking peptide (N-Terminus) or liposome ACKR1 protein, aiming at counteracting the potential effects of anti-ACKR1 antibodies. Liposome ACKR1 was used to preserve the structure of the antigen, especially the extracellular regions where antibodies selectively bind to. We examined the apoptosis of vein endothelial cells treated with pre-incubated IgG. In comparison with the vehicle control, plasma IgG alone did not induce necrotic and late apoptotic cells (Fig 3B), possibly attributable to the non-signaling nature of ACKR1 as a chemokine receptor. Likewise, both plasma IgG and anti-ACKR1 antibody control did not cause endothelial permeability in a transendothelial electrical resistance (TEER) assay (Fig 3C). Surprisingly, liposome ACKR1, but not blocking peptide, resulted in significantly reduced levels of necrotic and late apoptotic cells (Fig 3B), as well as improved endothelial barrier tightness as shown by increased TEER readings (Fig 3C). The protective effects of liposome ACKR1 could be because of internalization of liposomes via endocytosis mechanisms (34). Liposome internalization within endothelial cell lines underscore a predominant caveolae-dependent uptake, as evidenced in studies with human umbilical vein endothelial cells (35), alongside a partial involvement of clathrin-mediated uptake, particularly observed in human coronary artery endothelial cells (36). Furthermore, ACKR1 expressed by endothelial cells mitigates angiogenesis and inflammation, thereby restraining tumor growth (37). We postulated that the internalized ACKR1’s anti-angiogenic function stabilized endothelial barrier integrity and attenuated cell apoptosis.

Next, we investigated if anti-ACKR1 autoantibodies could impact on PBMC infiltration because ACKR1 is known to present chemokines on endothelial cells and facilitate leukocyte recruitment (15). In the endothelial-PBMC transwell co-culture assay, we observed no significance changes in PBMC transmigration across all the experimental groups (Fig 3D). It is possible that our experimental setup lacked chemokine transcytosis, a process necessary for generating chemokine gradients that guide leukocytes from the bloodstream to inflamed tissues in physiological contexts (16). Hence, we studied the treatment of purified IgG and/or PBMCs onto endothelial cells to evaluate direct antibody-dependent effects, immune cell-mediated responses, and antibody-dependent cellular cytotoxicity. We conducted a cell cytotoxicity assay based on the measurement of lactate dehydrogenase activity released from the cytosol of damaged endothelial cells into the supernatant. Interestingly, purified IgG or patient PBMCs led to significantly higher levels of antibody-dependent and immune cell-mediated cytotoxicity, respectively (Fig 3E). The introduction of both IgG and PBMCs pronouncedly enhanced antibody-dependent cellular cytotoxicity. Notably, the inclusion of blocking peptide or liposome ACKR1 to counteract the effects of anti-ACKR1 autoantibodies were effective in averting the extent of antibody-dependent cellular cytotoxicity (Fig 3F). In summary, our findings demonstrated that anti-ACKR1 autoantibodies could play a role in endothelial dysfunctions by augmenting antibody-dependent cellular cytotoxicity. The use of the blocking peptide might uncover that purified IgG from COVID-19 survivors possess potential binding sites for epitopes in N-terminal extracellular domain 1 of human ACKR1.

This marks the first report highlighting the role of anti-ACKR1 autoantibodies as drivers in subclinical vascular dysfunction. Our study unveils the presence of anti-ACKR1 autoantibodies associated with inflammatory risk, potentially predisposing individuals to cardiovascular diseases. These findings lay the foundation for more extensive cohort studies aimed at establishing the clinical significance of the long-term risk of vascular complications posed by anti-ACKR1 autoantibodies. This, in turn, suggests the potential for developing assays to identify individuals requiring more rigorous and targeted vascular-protective management. Moreover, the analysis of ACKR1 homologous sequences has deduced conservation of certain amino acid residues, but the highest divergence is observed in the N-terminal extracellular domain across various species (38). Undertaking further studies with a comprehensive panel for peptide screening to characterize epitopes can provide insights into the binding mechanisms and heterogeneities of human anti-ACKR1 autoantibodies. These will offer valuable information for the development of therapeutic modalities, including blocking peptides or neutralizing antibodies, with the goal of ameliorating specific autoantibody reactivity towards blood vessels in chronic inflammation. Finally, our findings lay the basis for extensive cohort studies to establish clinical significance of endothelial-targeting autoantibodies and identify individuals for more rigorous vascular-protective management.

## Materials and Methods

### Study approvals, patient enrolment, and sample collection

This study was approved by the Local Ethics Committee of the National Healthcare Group Domain Specific Research Board (2020/01426 and 2014/00236) and Nanyang Technological University Institutional Review Board (IRB-2020-09-011). Written informed consent was obtained from each participant after the nature, and possible consequences of the studies have been explained. The study protocol complies with the Helsinki Declaration.

This was part of a cross-sectional study investigating the long-term endothelial, hematological, and cardiovascular complications after recovery from COVID-19. Eligible participants, aged 21 yr and above, were identified among individuals admitted to the National Centre for Infectious Diseases, Singapore, between January 2020 and July 2021, with laboratory-confirmed SARS-CoV-2 infection determined by polymerase chain reaction. Inclusion criteria required patients to be between 6 and 15 mo post-recovery from COVID-19 infection. Baseline patient characteristics, comprising age, sex, ethnicity, comorbidities, and the severity and the month of COVID-19 infection, were collected through blood sampling at the time of enrolment (Table S1). The type of SARS-CoV-2 involved was determined based on the date of infection, and then categorized as Omicron, Delta, Pre-delta (Table S1).

For blood sample collection, 10 ml of fresh blood was collected from each participant via venepuncture and processed in the laboratory within 3 h. Upon Ficoll centrifugation of the blood specimen, the plasma fractions were preserved at −80°C for profiling of autoantibodies and cytokines. Buffy coat layers containing PBMCs were isolated for analysis of circulating endothelial cells and in vitro experimentation.

For the independent cohort, autoantibody profiling was performed using retrospective plasma samples stored at −80 °C from the EVAS study (39) and restricted to those consented for future studies. The samples analyzed in this study originated from patients with cardiometabolic risks (diabetes, hypertension, and hyperlipidaemia) but without prior established cardiovascular diseases (Table S2). Their clinical outcome measures were extracted from electronic medical record.

### Quantitative assessment of endothelial function by peripheral tonometry

We used the EndoPAT 2000 device (Itamar Medical Ltd) to assess the RHI (40). These assessments coincided with phlebotomy for fasting blood samples. Patients rested in a tranquil environment in a supine position at RT before the commencement of the procedures. Two flexible probes were positioned on the index fingers of both the right (occluded) and left (control) hands. Measurements were taken at baseline (6 min), during occlusion (5 min), and during reactive hyperemia (5 min) phases. The EndoPAT software automatically calculated RHI as the ratio of the average pulse wave amplitude during the baseline period in the occluded arm to the corresponding value in the control arm, adjusted by the baseline correction factor.

### Protein microarray for autoantibody detection

We custom made a microarray-based autoantibody detection kit (RayBiotech) where a glass slide surface was spotted with purified human recombinant ACKR1 protein (Cat #TP304680; OriGene) that could be recognized by ACKR1 autoantibodies present in human plasma. After we screened plasma samples, the arrays were incubated with biotin-conjugated anti-human IgG, after fluorescence dye-conjugated streptavidin. Bound plasma anti-ACKR1 autoantibodies, indicated by positive fluorescent signals would be captured with Agilent Surescan Microarray Laser Scanner, and quantitatively analyzed with Quantibody Q-Analyzer software. Plasma levels of anti-ACKR1 autoantibodies were calculated as normalized ratio of concentration against positive control, human IgG (50 μg/ml).

### Flow cytometry-based assay for detection of anti-ACKR1 antibodies

Protocol was adapted from reference 41. The preparation to generate an ACKR1-expressing cell line for the assay requires a human DARC cDNA clone (NCBI Accession # BC017817; Thermo Fisher Scientific). The complete open reading frame for ACKR1 gene was subcloned into a pWPXL-GFP transfer vector (derived from pWXL plasmid, kindly provided by Didier Trono, Ecole Polytechnique Fédérale de Lausanne, Lausanne, Switzerland). Lentiviral particles were produced and collected as previously described (42). K652 cells were transduced by adding the lentiviral particles in the supernatant. GFP-expressing K562 cells were sorted, expanded, and cryopreserved before the assay.

ACKR1 protein-expressing cells were seeded at 1.5 × 10^5^ cells per well in 96-well V-bottom plates. Cells were washed once with DPBS by centrifugation at 300*g* for 5 min. Cells were resuspended and incubated in diluted patient plasma (diluted 1:100 in FACS buffer [10% FBS in DPBS]) at 40°C for 30 min. Cells were washed twice with DPBS by centrifugation at 300*g* for 5 min. Then the cells were resuspended and incubated for a secondary stain with a double stain, consisting of Alexa Fluor 647-conjugated anti-human IgG (A21445, diluted 1:600; Thermo Fisher Scientific) and Zombie Aqua (423101, diluted 1:1,000; BioLegend) at 40°C for 30 min in the dark. Cells were washed twice with DPBS by centrifugation at 300*g* for 5 min. FACS buffer was added to the well to resuspend and cells were analyzed by flow cytometry immediately. Data analysis was performed with Flowjo.

### Profiling of circulating endothelial cells

PBMCs were subjected to staining in darkness for 10 min at RT, followed by 20 min at 4°C on an analog tube rotator with antibodies. Each subject’s sample used 1–3 million PBMCs, acquired from processed blood. After the incubation period, cells were rinsed and suspended in 200 μl of PBS containing 1% BSA for flow cytometry analysis. Circulating endothelial cells were identified based on the collective immunophenotypic markers of CD45−/CD31+/CD133−/DNA+. Details regarding antibodies can be found in the Supplemental Data 1. The quantification of circulating endothelial cells was represented as cells per million PBMCs. Flow cytometry analysis was performed using BD LSRFortessa X-20 (BD Biosciences) and FACSDiva software (BD Biosciences), whereas data interpretation used FlowJo v10.7.1 software (Becton Dickinson).

Supplemental Data 1. Antibodies used for flow cytometry and immunofluorescence staining.

### Quantitative PCR of endothelial antigens

Total RNA was isolated using RNeasy Plus Mini kit (QIAGEN) as per the manufacturer’s protocol, subsequently used to generate cDNA with LunaScriptTM RT SuperMix Kit (New England Biolabs). Realtime PCR was performed using SYBR green gene expression assays (New England Biolabs) on a QuantStudio 6 instrument (Applied Biosystems). Gene expressions were normalized to the endogenous GAPDH housekeeping gene. Please refer to Supplemental Data 2.

Supplemental Data 2. Primer sequences.

### Genotyping for ACKR1 phenotypes

ACKR1 polymorphism, G125A (rs12075) was genotyped to identify individuals carrying FYA (G125) and FYB (125A) alleles, defining Fy(a+b−), Fy(a−b+) and Fy(a+b+) phenotypes. As Fy(a−b−) phenotype is very rare in Asian populations (43), they were omitted from the analysis. Polymerase chain reaction (PCR) was performed under universal cycling conditions: 98°C for 10 min, followed by 35 cycles at 58.8°C for 45 s and 72°C for 1 min and with final enzyme denaturation at 72°C for 2 min, using Q5 High Fidelity DNA Polymerase (catalog no. M0491S; New England Biolabs). Amplified PCR products were run on a 1% agarose gel and the 825 base pair band was cut out and extracted using Monarch DNA Gel Extraction Kit (catalog no. T1020S; New England Biolabs). Individual ACKR1 genotypes were determined via Sanger sequencing (Bio Basic Asia). Please refer to Supplemental Data 2.

### IgG purification from patient plasma

Antibody Purification Kit (Protein A) (cat no. ab109209; Abcam) was prepared by coupling highly purified protein A to agarose beads and could be used to purify IgG fractions from pooled plasma samples (n = 5) of the highest anti-ACKR1 levels among the human subjects. The IgG were captured on the resin and unwanted substances were removed by a wash procedure following manufacturer’s manual. Purified IgG was then eluted and neutralized. The elution process was repeated three more times. Concentration of IgG fractions were determined using Bradford Protein Assay Kit (Cat no. A55866; Thermo Fisher Scientific) and calculated based on a BSA standard curve with a multiplication factor of 2.3 as recommended by manufacturer.

### Blocking of anti-ACKR1 autoantibodies

We interrogated the use of a liposome ACKR1 recombinant protein (cat # H00002532-G01; Abnova) or a blocking peptide (cat #LS-E30324; LSBio) which would bind and potentially negate the effect of anti-ACKR1 antibodies. ACKR1 is a seven-transmembrane glycoprotein comprising multiple known cytokine and antibody binding sites at four extracellular domains (33). The blocking peptide here is an 18 amino acid synthetic peptide near the N-Terminus, located within the first 50 amino acids (extracellular domain 1) of human ACKR1, where its major isoform is encoded by two common alleles, Fya (42Gly) and Fyb (42Asp). Another Fy6 epitope comprising amino acids residues 22-FEDVW-26 is also found within this region. Briefly, 10 μg/ml of purified IgG from patient plasma was pre-incubated with either 1 μg/ml of liposome ACKR1 protein or 1 μg/ml of blocking peptide at RT for 1 h before further experimentation on cell cultures.

### Apoptosis and viability assay with flow cytometry

Protocol for assessment of apoptosis and viability and was adapted from reference 44. Human umbilical vein endothelial cells were seeded at a cell density of 1 × 10^4^ cells/cm^2^ in EMG2 + 10% media and allowed to adhere overnight. On the treatment day, cells were 80% confluent. Cells were washed with DPBS (Cytiva) and treated for 24 h with 10 μg/ml of purified IgG, pre-incubated with or without 1 μg/ml blocking peptide or human ACKR1 recombinant protein expressed with a proteo-liposome (H00002532-G01; Abnova) for 1 h at RT.

On the apoptotic assay day, cells were washed with DPBS and dissociated with accutase (Gibco) for 5 min. Dissociated cells were centrifuged for 5 min at 300*g* in DPBS. Cell pellets were washed once with Annexin V binding buffer (422201; BioLegend) and resuspended in Annexin V binding buffer containing antibody including Annexin V Alexa Fluor 488 (1:40, A13201; Invitrogen) and Zombie Aqua (1:200, 423101; BioLegend). Cells were stained in the dark for 15 min at RT. Stained cells were washed once with Annexin V binding buffer and resuspended in Annexin V binding buffer and analyzed using BD LSRFortessa X-20 Cell Analyzer (BD Biosciences) immediately. Data analysis was performed with Flowjo.

### TEER measurements

TEER measurements were conducted using a WPI Epithelial Volt/Ohm (TEER) Meter (EVOM)2 (World Precision Instruments), following the manufacturer’s instructions. A total of 20,000 endothelial cells were seeded on 6.5 mm Transwell inserts with 5.0 μm polycarbonate membranes (3421; Corning). The next day, the cells were subjected to treatment with 10% pooled plasma in EGM2. TEER measurements were recorded at 24 h post-plasma treatment. Each treatment group was assessed in biological triplicates, and blank wells with no cells seeded were included as technical controls. TEER values (Ohms.cm^2^) were calculated using the formula: TEER = (resistance—average resistance of blank wells) x well surface area (0.33 cm^2^).

### Immune cell transmigration assay

A total of 20,000 human umbilical vein endothelial cells were seeded on the membranes of the transwell inserts, pore size of 5 μm (Corning), for 24 h. Concurrently, PBMCs were obtained from subjects from which IgG samples were pulled from and allowed to rest in RPMI 1640 supplemented with 10% FBS for 2 h. The seeded endothelial cells were treated with the different experimental conditions. PBMCs were then resuspended in an assay buffer of RPMI 1640 supplemented with 2% FBS, and seeded at 29,000 PBMCs per transwell. The PBMCs were allowed to transmigrate for 24 h and the numbers of transmigrated PBMCs to the bottom compartment were recorded.

### Antibody-dependent cell cytotoxicity assay

We used the Cytotoxicity Detection Kit (Cat. no. 11 644 793 001; Roche), a colorimetric assay for the quantification of cell lysis, based on the measurement of lactate dehydrogenase (LDH) activity released from the cytosol of damaged cells into the supernatant. Endothelial cells were seeded at a cell concentration of 1 × 10^4^ cells/200 μl culture media and allowed to adhere overnight. On the assay day, 10 μg/ml of purified IgG was pre-incubated with or without 1 μg/ml blocking peptide or liposome ACKR1 protein for 1 h. For IgG treatment on cells, endothelial cells were treated with 10 μg/ml of purified IgG at 37°C for 2 h. Meanwhile, frozen PBMCs (STEMCELL Technologies) were thawed and rested in RPMI media for 2 h at 37°C. To investigate antibody-dependent cell cytotoxicity, PBMCs (effector cells) were added to endothelial cells (target cells) in a ratio of 10:1, and co-cultured for 24 h. A “low” control was set up to determine spontaneous LDH activity from untreated endothelial cells, whereas a “high” control served as the maximum amount of releasable LDH enzyme activity by lysing the cells with 1% Triton X-100. Subsequently, cell-free supernatants were collected to determine the percentage cell-mediated cytotoxicity according to manufacturer’s manual. The average absorbance of triplicate samples and controls after subtracting the background from each, could be calculated in the following equation.

$$Cytotoxicity\;\left(\%\right)=\frac{(effector-target\;cell\;mix\;-\;effector\;cell\;control)\;-\;low\;control}{high\;control\;-\;low\;control}\times 100$$

### Statistical analysis

Statistical significance of differences was analyzed using GraphPad Prism version 9. Datasets with normal distributions were analyzed with one- or two-way ANOVA followed by post hoc Tukey for datasets with more than two conditions. Nonparametric Mann–Whitney *t* test was used for non-normally distributed data. The application of these statistical methods to specific experiments is noted in the figure legends. A *P-*value of < 0.05 was considered significant. The log-rank (Mantel-Cox) test and hazard ratio (log-rank) were used for the vascular disease incidence probability plot.

## Supplementary Material

Reviewer comments

## Data Availability

The authors declare that all data supporting the findings of this study are available within the article and supplemental materials. Further information and requests for resources and reagents should be directed to and will be fulfilled by the corresponding author. Some materials used in this study are commercially procured. There are restrictions to the availability of plasma and PBMC samples derived from human subjects because of ethics considerations for use of these materials within the current scope of the study. Requests can be made to the corresponding author as we will explore the use of materials subject to new ethics approval and research collaboration agreement (including material transfer).
