# Supplementary material for: Inflammatory risk contributes to post-COVID endothelial dysfunction through anti-ACKR1 autoantibody
Source: Life Sci Alliance. 2024 May 13;7(7):e202402598. doi: 10.26508/lsa.202402598 (PMC11091471; doi:10.26508/lsa.202402598)
Supplement: Supplementary file 1 [file LSA-2024-02598_TableS1.docx]

**Supplemental Materials**

**Table S1: Demographics of COVID-19 survivors and non-infected controls.**

| **Characteristics, N (%)** | **COVID-19 survivors**  **(n = 38)** | **Non-infected control**  **(n = 27)** |
| --- | --- | --- |
|  |  |  |
| Age [median (IQR)] | 43 (37, 55) | 55 (49.5, 60.5) |
| Gender, Male | 27 (71.1) | 20 (74.1) |
| Gender Female | 11 (28.9) | 7 (25.9) |
| Severity of COVID-19 | Asymptomatic: 11 (28.9)  Mild: 18 (47.4)  Oxygen supplementation: 9 (23.7) | N.A. |
| Type of type of SARS-CoV-2 infection | Omicron: 1 (2.6)  Delta: 9 (23.7)  Pre-delta: 28 (73.7) | N.A. |
| Days post hospitalization  [mean (± s.d.)] | 332 (96) | N.A. |
| **Cardiometabolic risk factors** | | |
| Hypertension | 8 (21.0) | 4 (14.8) |
| Hyperlipidemia | 8 (21.0) | 9 (33.3) |
| Diabetes mellitus | 11 (28.9) | 5 (18.5) |
| **Medical history** | | |
| Acute myocardial infarction | 2 (5.3) | 0 (0) |
| Chronic diabetic vasculopathy | 1 (2.6) | 0 (0) |
| Stroke | 0 (0) | 0 (0) |
| Venous thromboembolism | 0 (0) | 0 (0) |
| Fatty Liver | 1 (2.6) | 1 (5.9) |
| Autoimmune disease | 0 (0) | 0 (0) |
| Immunodeficiency | 0 (0) | 0 (0) |

*All values are reported as N (%) where N indicted number of observations.*
