# Supplementary material for: Inflammatory risk contributes to post-COVID endothelial dysfunction through anti-ACKR1 autoantibody
Source: Life Sci Alliance. 2024 May 13;7(7):e202402598. doi: 10.26508/lsa.202402598 (PMC11091471; doi:10.26508/lsa.202402598)
Supplement: Supplementary file 2 [file LSA-2024-02598_TableS2.docx]

**Supplemental Materials**

**Table S2: Demographics of independent cohort** **characterized by prevalent cardiometabolic risks without established cardiovascular diseases.**

| **Characteristics, N (%)** | **(n = 26)** |
| --- | --- |
|  |  |
| Age [median (IQR)] | 60.5 (56.5, 63.3) |
| Gender, Male | 12 (46.2) |
| Gender Female | 14 (53.8) |
| **Cardiometabolic risk factors** |  |
| Hypertension | 19 (73.1) |
| Hyperlipidemia | 23 (88.5) |
| Diabetes mellitus | 26 (100) |

*All values are reported as N (%) where N indicted number of observations.*
