# Supplementary material for: Inflammatory risk contributes to post-COVID endothelial dysfunction through anti-ACKR1 autoantibody
Source: Life Sci Alliance. 2024 May 13;7(7):e202402598. doi: 10.26508/lsa.202402598 (PMC11091471; doi:10.26508/lsa.202402598)
Supplement: Supplemental Data 1. — Antibodies used for flow cytometry and immunofluorescence staining. [file LSA-2024-02598_Supplemental_Data_1.docx]

**Supplemental Materials**

**Antibodies used for flow cytometry and immunofluorescence staining.**

| **Antibodies** | **Dilution** | **Manufacturer,**  **catalogue number** | **RRID** | **Purpose** |
| --- | --- | --- | --- | --- |
| BD Pharmingen™ PE Mouse Anti-Human CD234 (DARC) | 5ul/million cells | BD Biosciences, 566424 | AB_2739724 | ACKR1 expression in flow cytometry; positive control for TEER and cytotoxicity assay |
| Hoechst 33342 Ready Flow™ Reagent | 5 µl/million cells | Thermo Fisher Scientific  R37165 |  | Circulating endothelial cell detection |
| PE/Cyanine7 anti-human CD31 Antibody | 4 µl/million cells | BioLegend  303118 | AB_2247932 |  |
| APC anti-human CD133 Antibody | 4 µl/million cells | BioLegend  397906 | AB_2876721 |  |
| PE anti-human CD45 Antibody | 3 µl/million cells | BioLegend  368510 | AB_2566370 |  |
| Goat anti-human IgG AF647 | 1:1000 | Thermo Fisher Scientific,  A-21445 | AB_2535862 | Anti-ACKR1 antibody detection in Flow Cytometry |

| **Other Reagents** | **Manufacturer,**  **catalogue number** | **RRID / ORF** | **Purpose** |
| --- | --- | --- | --- |
| Recombinant Human TNF-α | PeproTech, 300-01A |  | Fig 1A |
| DARC (Human) Recombinant Protein (liposome ACKR1) | Abnova, H00002532-G01 | NP_002027.2 | Fig 3 |

| **Cells** | **Manufacturer,**  **catalogue number** | **Lot Number** | **Purpose** |
| --- | --- | --- | --- |
| Human Umbilical Vein Endothelial Cells (HUVEC) | Lonza, C2519A | Lot#21TL347550 | Fig 1 and 3 |
| Human Umbilical Artery Endothelial Cells (HUAEC) | Promocell, C-12202 | Lot# 436Z013.2 | Fig 1 |
