# Supplementary material for: Inflammatory risk contributes to post-COVID endothelial dysfunction through anti-ACKR1 autoantibody
Source: Life Sci Alliance. 2024 May 13;7(7):e202402598. doi: 10.26508/lsa.202402598 (PMC11091471; doi:10.26508/lsa.202402598)
Supplement: Supplemental Data 2. — Primer sequences. [file LSA-2024-02598_Supplemental_Data_2.docx]

**Supplemental Materials**

**Primer sequences**

| **Genes** | **Forward (5' - 3')** | **Reverse (3' - 5')** | **Remarks** |
| --- | --- | --- | --- |
| ***GAPDH*** | CCATCTTCCAGGAGCGAGA | GATGACCCTTTTGGCTCCCC | Figure 1A |
| ***ACKR1*** | CCTGAACCAAACGGTGCCAT | GCACCATAGTCTCCATCTGGG |  |
| ***RSPO3*** | TAGTCAAGGCTGCCAAGGAG | TCACAGTCAGCTTTGCATTTTGT |  |
| ***ACVRL1*** | CACGACAACATCCTAGGCTTCA | CAGAAAGTCGTAGAGGGAGCC |  |
| ***MADCAM1*** | AGCTCCTTGTGTACGCCTTC | ACGAGCAGGGAGAAGGAGAG |  |
| ***ICAM1*** | GGAGCTTCGTGTCCTGTATGG | CAGTGGGAAAGTGCCATCCT |  |
| ***ACKR1***  rs12075 DNA sequencing Duffy allele | ATGGCCTCCTCTGGGTATGT | CAACAGCAACAGCTTGGACC | Figure S2B |
